# Dichlorine–pyridine *N*-oxide halogen-bonded complexes†

**DOI:** 10.1039/d4sc06270a

**Published:** 2024-10-21

**Authors:** Niklas Limberg, J. Mikko Rautiainen, Jan Lundell, Sebastian Riedel, Kari Rissanen, Rakesh Puttreddy

**Affiliations:** a Department of Chemistry and Biochemistry, Freie Universität Berlin Fabeckstr. 34/36 14195 Berlin Germany s.riedel@fu-berlin.de; b Department of Chemistry, University of Jyvaskyla P.O. BOX 35 FI-40014 Jyväskylä Finland kari.t.rissanen@jyu.fi rakesh.r.puttreddy@jyu.fi

## Abstract

A new Cl–Cl···^−^O–N^+^ halogen-bonded paradigm has been demonstrated, using dichlorine as a halogen bond (XB) donor and *N*-oxide as an XB acceptor. Their crystalline complexes were formed during the warm-up process from −196 °C to −80 °C for X-ray diffraction analysis. They exhibit high instability in the crystalline state, even at these low temperatures, leading to rapid decomposition and the formation of Cl⋯H–O–N hydrogen-bonded complexes. The normalized XB interaction ratio (*R*_XB_) of Cl⋯O interactions in the solid-state demonstrates affinity comparable to traditional I⋯O interactions observed in I–I···^−^O–N^+^ halogen-bonded systems. The Cl–Cl⋯O XB angles vary from 172° to 177°, manifesting the structure-guiding influence of the electronegative chlorine atom's σ-hole on these XB interactions.

## Abstract 2

The first structural evidence of extremely unstable Cl⋯O halogen bonds formed by dichlorine and pyridine *N*-oxide has been successfully characterized by X-ray diffraction analysis.

## Graphical abstract

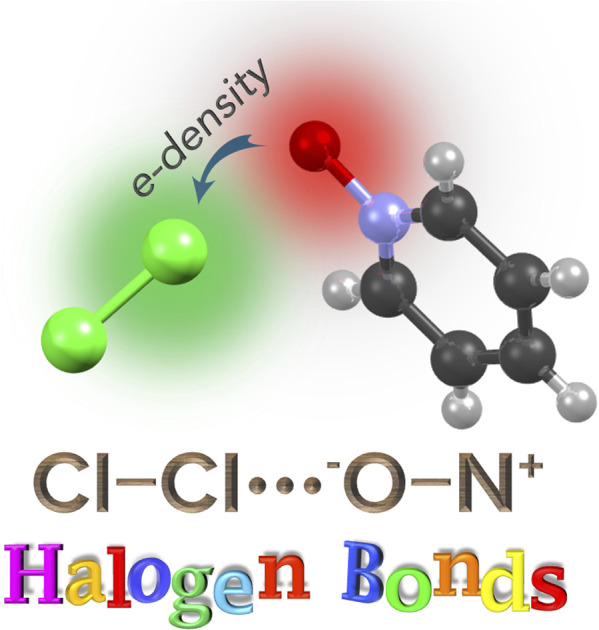

## Introduction

**Fig. 1:**
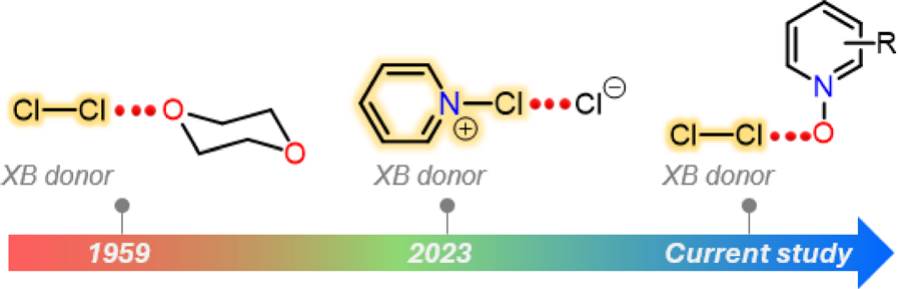
X-ray crystal structures of Cl⋯O/N halogen-bonded complexes reported to date.

Halogen bonding is an attractive interaction of the type R–X⋯B, where X is a halogen atom, and B can be any kind of Lewis base (*e.g.*, N, O, and S).^1^ The basis of this interaction is the occurrence of an anisotropic charge distribution around the X-atom, which leads to the formation of a so-called σ-hole, a region of decreased electron density on the extension of a R–X bond.^2^ The size of the σ-hole has been shown to define the directionality of the XB interaction. This preference was explained by natural bond order analysis of alkyl halides by Clark *et al.*, who described an approximate s^2^p_*x*_^2^p_*y*_^2^p_*z*_^1^ configuration (where *z* is the orientation of the R–X bond) and a deficient electron density site on the halogen atom.^3^ In general, the heavier iodine and bromine atoms possess larger σ-holes than the lighter chlorine and fluorine atoms enabling the heavier halogen atoms to more effectively accept electron density from a Lewis base. In view of an MO picture the Lewis base donates electron density to a σ* orbital of R−X species. This characteristic feature has been exploited in a palette of I/Br⋯N/O/S XBs for a wide range of applications from crystal engineering to biology.^4–6^ In contrast, the CI⋯B XBs were exclusively studied using computational approaches.^7–9^ Their X-ray crystallography data are extremely rare.^10,11^ In 1950s, Hassel and Strømme solved the X-ray crystal structure of the 1 : 1 Cl_2_·dioxane^11^ adduct featuring Cl⋯O contacts (*ca*. 2.678 Å) that are below the sum of the van der Waals radii of Cl- and O-atoms (3.27 Å), indicating an attractive interaction. Almost 70 years later, we have reported the X-ray crystal structures of a series of Cl⋯N XB complexes, including [pentafluoropyridine-Cl]^+^[AsF_6_]^−^, [bis(pyridine)Cl]^+^[BF_4_]^−^, [(lutidine-Cl)^+^](lutidine)[Cl_3_]^−^ and a 1 : 1 [(pyridine-Cl)^+^][Cl]^−^ adduct (Fig. 1).^10^ Notably, in all these complexes, the Cl-atom resides extremely close to the pyridinic nitrogen, with Cl⋯N distances ranging between 1.754(2) and 2.232(6) Å.

Pyridine *N*-oxides (PyNOs) are versatile ambivalent species, allowing them to react with both nucleophilic and electrophilic reagents.^12^ Consequently, they have received recognition as highly valuable synthetic intermediates in the preparation of pyridines.^13^ Despite their lower basicity compared to pyridines, their ability to activate various Lewis acids is often adequate to catalyze numerous organic reactions. Thus, the research in PyNO chemistry has centred on organic synthesis, leaving their potential underexplored in supramolecular chemistry. In the context of XB chemistry, considerable efforts have been made in understanding the I⋯O XBs formed by perfluoroiodoalkanes/aromatics and PyNOs by the research groups of Resnati,^14,15^ Rosokha,^16,17^ Jin,^18–20^ and Pennington.^21^ Although less common, reports of Br⋯O XBs^22,23^ involving aromatic XB donors and PyNOs also exist. Apart from the above mentioned XBs, complexes involving PyNOs and dihalogens of the type X_2_·PyNO (X = I) are exclusively known with iodine and have been studied by X-ray crystallography.^16,18,24^ In contrast, the high affinity of *N*-oxides for the water content in the atmosphere has hampered the isolation of X_2_·PyNO (X = Br, Cl, F) complexes for X-ray diffraction studies. For instance, attempts to isolate the highly reactive Br_2_·PyNO have typically resulted in the formation of [PyNO-H]^+^[Br]^−^ and [(PyNO)_2_-H]^+^[Br]^−^ type hydrogen-bonded complexes^25,26^ as a result of the reaction with the solvent molecules. Therefore, there is an overwhelming lack of knowledge about the chemistry of X_2_·PyNO (X = Br, Cl, F) complexes in the solid state.

Here, we report the synthesis, X-ray crystallography, and Density Functional Theory (DFT) studies of dichlorine–PyNO complexes, demonstrating the potential of electronegative chlorine's σ-hole in forming strong Cl–Cl···^−^O–N^+^ XBs comparable in strength to I–I···^−^O–N^+^ XBs.

## Results and discussion

**Fig. 2:**
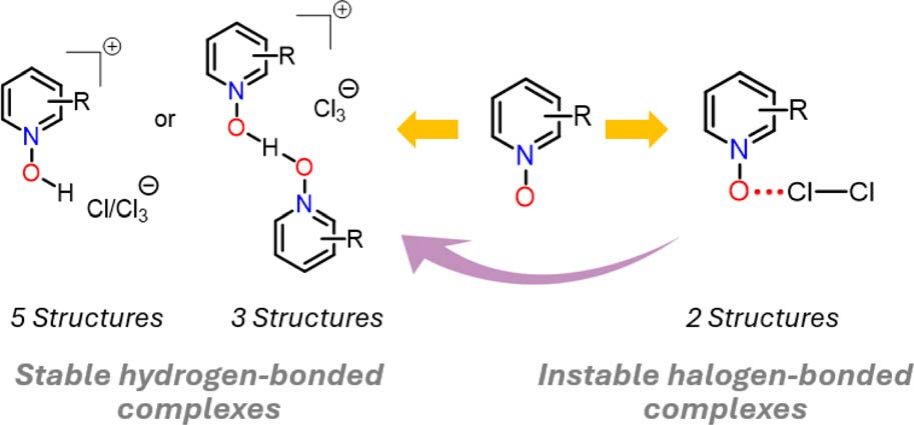
A summary of X-ray crystal structures of halogen- and hydrogen-bonded complexes.

The dichlorine–PyNO halogen-bonded complexes are formed by condensing one millimole of elemental Cl_2_ onto one millimole of appropriate PyNO dissolved in propionitrile at −196 °C in a Schlenk tube with a PTFE valve. The solutions were warmed to −80 °C and maintained at that temperature to form crystals suitable for X-ray diffraction analysis. Despite our rigorous attempts to broaden the scope, only two crystal structures, Cl_2_-PyNO and Cl_2_-26DiMePyNO (26DiMePyNO = 2,6-dimethylpyridine *N*-oxide) could be isolated and characterized by X-ray diffraction analysis. Due to the polydentate coordination mode of *N*-oxide oxygen and its strong affinity for protonation and formation of hydrogen-bonded complexes, the Cl⋯O XB complexes exhibited high instability, transforming into hydrogen-bonded complexes of the type [PyNO-H]^+^[Cl]^−^ and [(PyNO)_2_-H]^+^[Cl_3_]^−^ (Fig. 1 and S1–S8†). For instance, when the Schlenk tube containing Cl_2_-PyNO and Cl_2_·(26DiMePyNO) complexes was opened in an argon environment to select crystals for X-ray crystallography, their bulk samples immediately reacted with water traces from air to form [PyNO-H]^+^[Cl]^−^ and [26DiMePyNO-H]^+^[Cl]^−^ complexes, respectively (Fig. 2). Furthermore, it is important to note that the chlorine complex of 3-methoxypyridine *N*-oxide exhibited strong reactivity during the warming process from −196 to −80 °C, resulting in shattering of the Schlenk tubes. These aspects prompted us to limit our current investigation to X-ray crystallography of two XB complexes and DFT studies.

**Fig. 3:**
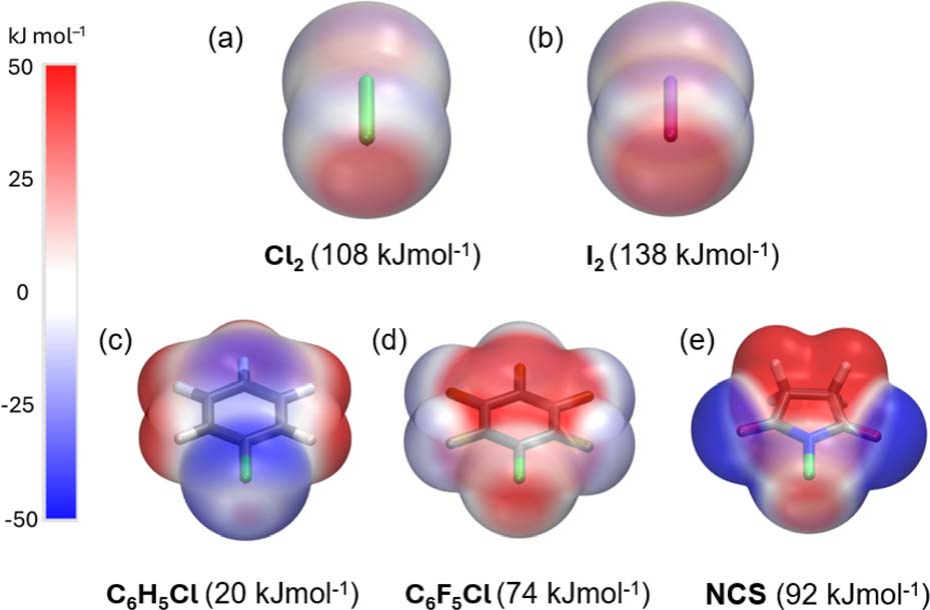
Computed electrostatic potential surface (ESP) at PBE0-D3/def2-TZVP level projected on the 0.001 au electron density surfaces of XB donors with *V*_S,max_ values, (a) Cl_2_, (b) I_2_, (c) C_6_H_5_Cl, (d) C_6_F_5_Cl, and (e) NCS.

Electrostatic potential (ESP, *V*_s,max_) maps were computed at the PBE0-D3/def2-TZVP^27–35^ level of theory (Fig. 3a–e) to compare the σ-hole strength of Cl_2_ with a more common I_2_ XB donor. Despite the large electronegativity differences, the σ-hole strength of dichlorine and diiodine differs only by 30 kJ mol^−1^. Furthermore, the *V*_s,max_ for Cl_2_ (+108 kJ mol^−1^) exceeds that of aromatic XB donors, *e.g.*, C_6_H_5_Cl (+20 kJ mol^−1^) and C_6_F_5_Cl (+74 kJ mol^−1^), as well as non-aromatic *N*-chlorosuccinimide (NCS, +92 kJ mol^−1^). The *V*_s,max_ values follow the XB donor strength order: I_2_ > Cl_2_ > NCS > C_6_F_5_Cl > C_6_H_5_Cl, suggesting that Cl_2_ is the strongest XB donor among the Cl-donors from an electrostatic perspective.

**Fig. 4:**
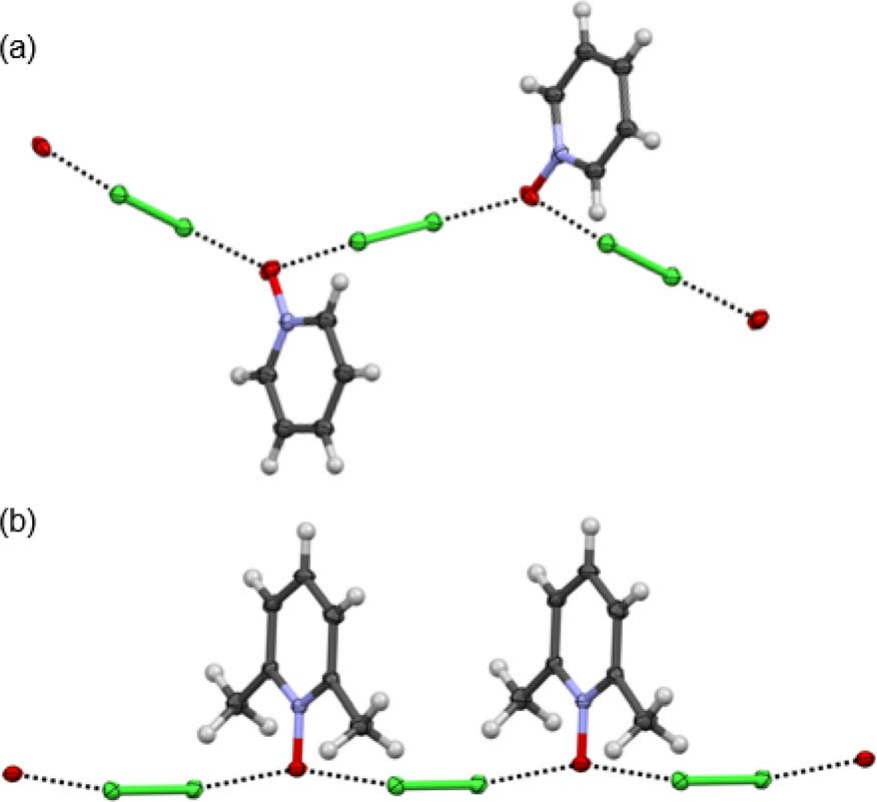
The X-ray crystal structures of (a) Cl_2_-PyNO and (b) Cl_2_-26DiMePyNO with the thermal displacement parameter at the 50% probability level. Disordered parts in Cl_2_-PyNO are omitted for clarity.

The 1 : 1 donor : acceptor complexes of Cl_2_-PyNO and Cl_2_-26DiMePyNO are shown in Fig. 4a and b. The asymmetric unit of Cl_2_-PyNO consists of two molecules of Cl_2_ and two molecules of PyNO, whereas the asymmetric unit of Cl_2_-26DiMePyNO consists of half a molecule of Cl_2_ and a full 26DiMePyNO. Their packing structures revealed that both complexes form infinite 1D polymeric chains *via* short Cl⋯O XB contacts (Fig. 1). The bidentate *N*-oxide oxygen atoms connect the dichlorine molecules. The interatomic distances between Cl and O atoms in Cl_2_-PyNO range from 2.567(5) Å to 2.627(5) Å, while in Cl_2_-26DiMePyNO, it is 2.5676(8) Å.

Their normalized interaction ratio (*R*_XB_)^36,37^ values are between 0.79–0.80. Notably, the *R*_XB_ values of Cl⋯O contacts in Cl_2_-PyNO are similar to those of I⋯O interactions (2.684(8)–2.791(8) Å, *R*_XB_ = 0.77–0.80) in the reported l_2_-PyNO complex.^17^ The Cl–Cl⋯O angles vary between 171.98(5)° and 177.09(18)°. These characteristics collectively indicate a strong interaction between chlorine and *N*-oxide oxygen in the form of XBs.

**Fig. 5:**
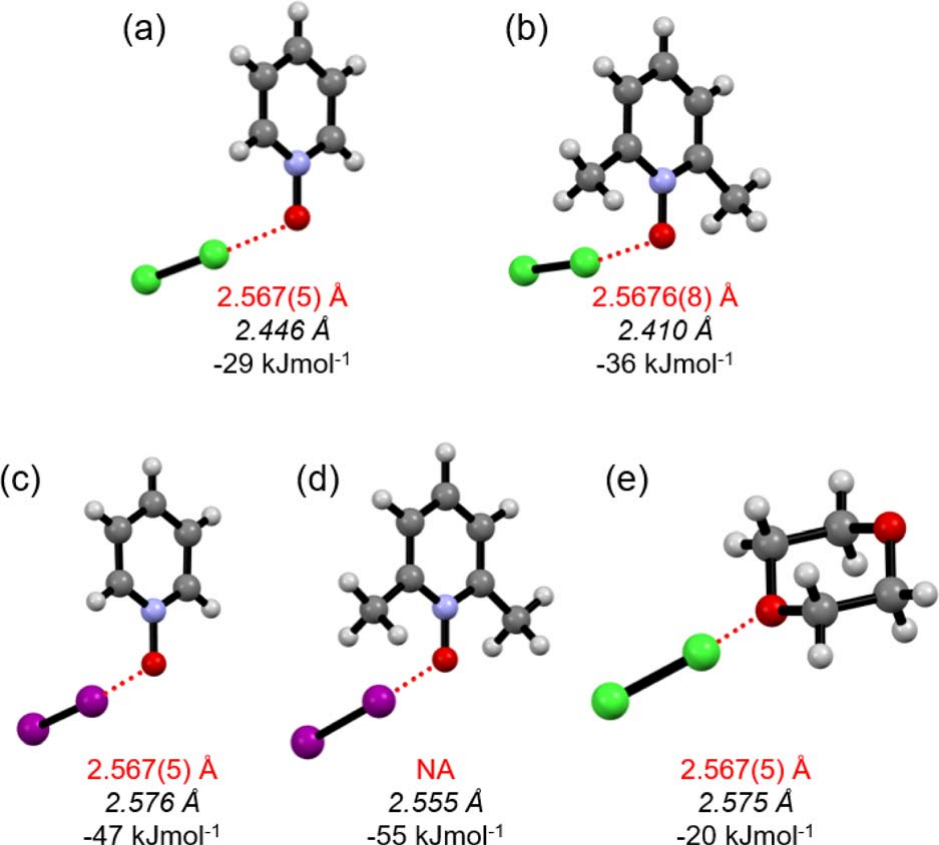
The DFT structures of (a) Cl_2_-PyNO (b) Cl_2_-26DiMePyNO, (c) I_2_-PyNO (d) I_2_-26DiMePyNO and (e) Cl_2_-dioxane optimized at the PBE0-D3/def2-TZVP level of theory. Red broken lines represent XBs. The red font represents XB distances of the crystal structure and the italicized black font represents XB distances of DFT optimized structures. NA: crystal structure is not available.

The Cl–Cl···^−^O–N^+^ XB interaction energies (Δ*E*_XB_) in Cl_2_-PyNO and Cl_2_-26DiMePyNO complexes with a 1 : 1 donor : acceptor ratio were calculated and compared with the I–I···^−^O–N^+^ energies in l_2_-PyNO and l_2_-26DiMePyNO complexes, respectively (Table S1†). The optimized Cl⋯O distances in Cl_2_-PyNO and Cl_2_-26DiMePyNO are 2.446 and 2.410 Å, respectively, which are 0.152 Å and 0.158 Å shorter than those observed in their crystal structures. The Δ*E*_XB_ values of Cl⋯O contacts are smaller than those of I⋯O XBs, with the Δ*E*_XB_ values of Cl/I···^−^O–N^+^ XBs of unsubstituted PyNO complexes being smaller than those of 26DiMePyNO complexes (Fig. 5). This difference can be attributed to the larger nucleophilic character of the 26DiMePyNO's oxygen due to the electron-donating *ortho*-methyl groups. In comparison to the Cl⋯O XBs in the reported Cl_2_-dioxane^11^ complex, which has an energy of −20 kJ mol^−1^, the Cl···^−^O–N^+^ interaction energies are larger, varying between −29 and −36 kJ mol^−1^. This can be attributed to the efficient overlap of the negatively charged *N*-oxide oxygen atom lone pair and p-orbital of the donor chlorine as compared to the uncharged ether oxygen atom.

The protonated *N*-oxide stabilized by the chloride or trichloride counter anion is the common and stable outcome of these crystallizations. Attempts to model the Cl⋯H–^−^O–N^+^ hydrogen-bonded structures of [PyNO-H]^+^[Cl]^−^ and [(26DiMePyNO)-H]^+^[Cl]^−^ in the gas phase resulted in non-charge separated 1 : 1 adducts of HCl and PyNO and HCl and 26DiMePyNO with Cl–H···^−^O–N^+^ energies of −59 and −71 kJ mol^−1^, respectively. The Cl–H···^−^O–N^+^ energies are roughly twice as high as the Cl–Cl···^−^O–N^+^ XB interaction energies. An estimation of the Cl^−^···H^+^–^−^O–N^+^ interaction energy can be made by single point calculation of the [(26DiMePyNO)-H]^+^[Cl]^−^ in the crystal structure environment showing −435 kJ mol^−1^ as the interaction energy between charged species.

The ^15^N nitrogen chemical shifts in nitrogen-metal/halogen-bonded complexes are lower compared to their non-coordinated forms. The lower ^15^N nitrogen chemical shifts in complexes can be attributed to the decrease in the paramagnetic deshielding term on the pyridinic nitrogen during metal^38,39^/halogen-bond^40,41^ complexation, caused by the replacement of n–π* electron transitions with σ–π* transitions.^42,43^ In principle, this can be experimentally determined by ^15^N NMR spectroscopy by estimating the coordination shift Δ*δ*(^15^N), which is the difference in *δ*(^15^N) chemical shift between a complex (*δ*^15^N_compl_) and its corresponding pyridine ligand (*δ*^15^N_lig_). However, owing to the difficulties in handling dichlorine-PyNO samples for NMR studies, we used the DFT calculations to analyse ^15^N NMR chemical shifts of *N*-oxides in their uncomplexed and complexed forms to estimate Δ*δ*(^15^N) values (Table 1 and S2†). The isotropic shielding constants were computed at the relativistic level using the ZORA–PBE0/TZ2P^46,47^ method using a COSMO^48^ model to treat the solvation effects. The chemical shifts were determined by calibrating the NIS shift to the experimental shift and scaling the chemical shifts of *N*-oxide ligands and their complexes correspondingly. To determine the coordination power of *N*-oxides in Cl_2_-PyNO and Cl_2_-26DiMePyNO, their ^15^N NMR chemical shifts were compared to the calculated ^15^N chemical shifts of I_2_-PyNO, I_2_-26DiMePyNO, NIS-PyNO and NIS-26DiMePyNO.

**Table 1:** DFT ^15^N NMR chemical shifts and ^15^N coordination shifts [in ppm] calculated with the ZORA–PBE0/TZ2P method

	^15^N Chemical shift (ppm)	Δ*δ*(^15^N) = *δ*^15^N_compl_ − *δ*^15^N_lig_ (ppm)
PyNO	−90 (−90)^a^	—
26DiMePyNO	−95 (−95)^a^	—
Cl_2_-PyNO	−106	−16
Cl_2_-26DiMePyNO	−111	−16
I_2_-PyNO	−111	−21
I_2_-26DiMePyNO	−116	−21
NIS-PyNO	−110	−20 (−9)^a^
NIS-26DiMePyNO	−116	−21 (−13)^a^

a The values in parentheses represent experimental ^15^N NMR data acquired in CDCl_3_ (ref. 44 and 45)

The ^15^N chemical shifts of PyNO and 26DiMePyNO are −90 ppm and −95 ppm, respectively. Additionally, their chlorine and iodine/NIS complexes exhibit shifts ranging between −106 and −116 ppm (Table 1). The observed trend in Δ*δ*(^15^N) values indicates similar Δ*δ*(^15^N) values for both PyNO and 26DiMePyNO complexes, regardless of the XB interaction type. The difference in coordination powers suggested by the Δ*E*_XB_ values of PyNO and 26DiMePyNO complexes appears not to be significant enough to affect the Δ*δ*(^15^N) values. The magnitude of the Δ*δ*(^15^N) values of chlorine complexes is slightly smaller than that of the iodine/NIS complexes, indicating that the Cl–Cl⋯^−^O–N^+^ halogen-bonded systems formed by Cl_2_ and *N*-oxides demonstrate the n(O) → σ*(Cl) electron donation strength almost to the same extent as the N–I/I–I⋯^−^O–N^+^ systems. The comparison between this small set of solid-state and DFT results and our comprehensive study on N/C−I⋯^−^O–N^+^ XBs^44,49^ suggests that the structure directing element of the Cl⋯O interactions is the chlorine σ-hole.

## Experimental

### Materials

Pyridine *N*-oxide (>95%) was purchased from Sigma-Aldrich, whereas 3-phenylpyridine *N*-oxide, 3-methoxypyridine *N*-oxide, 2-methyl-4-nitropyridine *N*-oxide, 2,6-dimethylpyridine *N*-oxide, 2,4-dimethylpyridine *N*-oxide, 2-methyl-4-methoxy pyridine *N*-oxide, 2,4,6-trimethylpyridine *N*-oxide, and 4-*tert*-butylpyridine *N*-oxide were synthesized using the literature method.^50^ Chlorine was purchased from Linde. Propionitrile (>90%) was purchased from Sigma-Aldrich. Prior to crystallization experiments, the *N*-oxides were vacuum-dried overnight, and SPS propionitrile was dried over 4 Å molecular sieves.

### X-ray crystallography

The X-ray crystal structure data of all crystal structures were collected at 100 K, using a Bruker D8 Venture diffractometer equipped with a CMOS area detector and Mo-K_α_ (*λ* = 0.71073 Å) radiation. APEX5 (version v2023.9-2) was used for the data collection and reduction. The intensities were absorption corrected using a multi-scan absorption correction method. All structures were solved by intrinsic phasing (SHELXT)^51^ and refined by full-matrix least squares on *F*^2^ using the OLEX2 (ref. 52) and utilizing the SHELXL-2015 (ref. 51) module. Anisotropic displacement parameters were assigned to non-H atoms and isotropic displacement parameters for all H atoms were constrained to multiples of the equivalent displacement parameters of their parent atoms with *U*_iso_(H) = 1.2 *U*_eq_(parent atom).

### DFT calculations

To be consistent with our earlier halogen bonding studies on *N*-oxide systems,^37,44^ DFT calculations were carried out with the PBE0 hybrid functional^28,30,31^ employing def2-TZVP basis sets^34,35^ and treating dispersion interactions with the empirical D3 model by Grimme that includes Becke–Johnson damping.^32,33^ The counterpoise method was used to derive basis set superposition error corrected complexation energies.^53^ The Gaussian 16 program package^54^ was used for DFT optimizations. Calculations for isotropic ^15^N chemical shielding constants^55^ were carried out with the ADF 2022.1 program^56^ at the relativistic two-component ZORA^46,47^ level using the PBE0 hybrid functional and TZ2P^57^ basis sets. The chloroform solvent (dielectric constant *ε* = 4.81) environment was simulated using the implicit COSMO^48^ solvent model (for more details, see the ESI†).

### Synthesis of dichlorine–*N*-oxide complexes

#### Cl_2_-PyNO

The *N*-oxide (232 mg, 2.4 mmol, 1.0 eq.) was dissolved in propionitrile (7.0 mL) in a Schlenk tube and cooled to −196 °C. Chlorine gas (172 mg, 2.4 mmol, 1.0 eq.) was then added to the frozen solid mass, which was allowed to warm to −80 °C inside a fume hood and then transported at that temperature to a −80 °C freezer. After 5 days, the pale yellowish solution yielded colourless crystals. Note: When the Schlenk was opened at −80 °C under a nitrogen atmosphere to select crystals for X-ray diffraction analysis, the crystals dissolved.

#### Cl_2_-26DiMePyNO

The *N*-oxide (107 mg, 0.87 mmol, 1.0 eq.) was dissolved in propionitrile (7.0 mL) in a Schlenk tube and cooled to −196 °C. Chlorine gas (62 mg, 0.87 mmol, 1.0 eq.) was then added to the frozen solid mass, which was allowed to warm to −80 °C inside a fume hood and then transported at that temperature to a −80 °C freezer. After one week, the light-yellow solution yielded colourless crystals. Note: when the Schlenk was opened at −80 °C under a nitrogen atmosphere to select crystals for X-ray diffraction analysis, the crystals dissolved.

Caution! The reaction mixtures can react violently during the warming process from −196 to −80 °C. Care must be taken when handling the samples.

## Conclusions

The combined findings from X-ray crystallography and theoretical investigations of dichlorine-pyridine *N*-oxide and dichlorine-2,6-dimethylpyridine *N*-oxide halogen-bonded complexes indicate that dichlorine serves as a moderately strong XB donor and it's σ-hole exerts a guiding influence on the halogen-bonded systems. The unsubstituted pyridine *N*-oxide forms a weaker Cl⋯O interaction (−29 kJ mol^−1^) in contrast to the 2,6-dimethylpyridine *N*-oxide (−36 kJ mol^−1^) with two electron-donating methyl groups proximal to the N–O group. The Cl–CI···^−^O–N^+^ halogen-bonded systems exhibit structural and energetic similarities to I–I···^−^O–N^+^ systems and comparable energy levels to (imide) N–I···^−^O–N^+^ systems, yet the chlorine complexes display high instability. Thus, broadening the Cl⋯O scope, while highly desirable, poses a formidable challenge. Nevertheless, the role of the σ-hole on the electronegative chlorine should not be underestimated when investigating interactions between chlorinated species and Lewis bases.

## Author contributions

R. P. designed the project and wrote the manuscript. R. P. and N. L. carried out the crystallization experiments and chlorination reactions. N. L. did the X-ray crystallography. J. M. R. carried out the computational studies. All the authors discussed the results and commented on the manuscript.

## Conflicts of interest

There are no conflicts to declare.

## Supplementary Material

SC-015-D4SC06270A-s001

SC-015-D4SC06270A-s002

SC-015-D4SC06270A-s003

## Data Availability

The ESI data† for this article include X-ray crystallography data and DFT optimized structures as xyz files. Crystal structures are deposited in The Cambridge Crystallographic Data Centre (CCDC). Deposition numbers 2383807 (for Cl_2_-PyNO), 2383799 (for Cl_2_-26DiMePyNO), 2383800 (for [PyNOH][Cl]), 2383803 (for H[2PhPyNO][Cl_3_]) 2383804 (for H[246TriMePyNO]_2_[Cl_3_]), 2383802 (for H[24DiMePyNO]_2_[Cl_3_]·2[24DiMePyNO]), 2383801 (for H[2Me_4_OMePyNO]_2_[Cl_3_]), 2383798 (for [26DiMePyNOH][Cl]), 2383806 (for [2Me_4_OMePyNOH][Cl]), and 2383805 (for [4tBuPyNOH][Cl]) contain the ESI† crystallography data for this paper.
