# Supplementary material for: Dichlorine–pyridine N-oxide halogen-bonded complexes
Source: Chem Sci. 2024 Oct 21;15(45):18840–5. doi: 10.1039/d4sc06270a (PMC11526815; doi:10.1039/d4sc06270a)
Supplement: SC-015-D4SC06270A-s001 [file SC-015-D4SC06270A-s001.pdf]

Supplementary Information (SI) for Chemical Science.
This journal is © The Royal Society of Chemistry 2024

Supporting Information

Niklas Limberg,[a] J. Mikko Rautiainen,[b] Jan Lundell,[b] Sebastian Riedel,[a] Kari Rissanen,[b] Rakesh Puttreddy[b]

[a]*Department of Chemistry and Biochemistry, Freie Universität Berlin, Fabeckstr. 34/36, 14195 Berlin, Germany.*

[b]*University of Jyvaskyla, Department of Chemistry, P.O. Box. 35, FI-40014 University of Jyvaskyla, Finland*

Table of Contents

1. X-ray Crystallography ***Page S2***

2. Density Functional Theory Studies (DFT) ***Page S7***

1. X-ray Crystallographic Studies

1.1 General information

The X-ray crystal structure data for **H[246TriMePyNO]₂[Cl₃] - Cl₂-26DiMePyNO** were collected at 100 K, using a Bruker D8 Venture diffractometer equipped with a CMOS area detector and Mo-K_α (λ = 0.71073 Å) radiation. APEX5 (version v2023.9-2) was used for the data collection and reduction, and the intensities were absorption corrected using a multi-scan absorption correction method. All structures were solved by intrinsic phasing (SHELXT)[2] and refined by full-matrix least squares on F^2 using the OLEX2[3], utilizing the SHELXL-2015 module.[4,5] Anisotropic displacement parameters were assigned to non-H atoms and isotropic displacement parameters for all H atoms were constrained to multiples of the equivalent displacement parameters of their parent atoms with U_{iso}(H) = 1.2 U_{eq}(parent atom). The X-ray single crystal data and CCDC numbers of all new structures are included below. CCDC 2383798-2383807 contains the supplementary crystallographic data for this paper. These data can be obtained free of charge via http://www.ccdc.cam.ac.uk/conts/retrieving.html (or from the CCDC, 12 Union Road, Cambridge CB2 1EZ, UK; Fax: +44 1223 336033; E-mail: deposit@ccdc.cam.ac.uk).

1.2 Hydrogen-bonded complexes

1.2.1 Complex H[246TriMePyNO]$_2$[Cl$_3$]

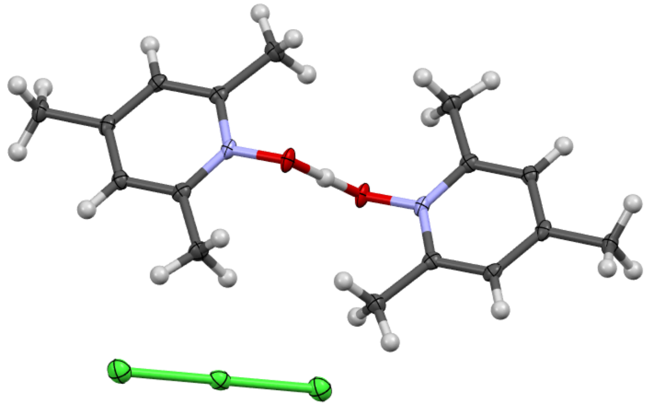

Figure S1. The X-ray crystal structure of **H[246TriMePyNO]$_2$[Cl$_3$]** with the thermal displacement parameter at 50% probability level. Colour Key: green = chlorine, blue = nitrogen, dark grey = carbon, white = hydrogen.

Crystal data for **H[246TriMePyNO]$_2$[Cl$_3$]**: CCDC-2383804. $C_{16}H_{23}Cl_3N_2O_2$, M = 381.71 g/mol, clear yellow block, 0.235 × 0.199 × 0.177 mm^3, triclinic, space group $P\bar{1}$, a = 7.2228(13) Å, b = 8.5908(16) Å, c = 8.6321(17) Å, α = 68.449(6)°, β = 65.454(6)°, γ = 78.430(7)°, V = 452.42(15) Å^3, Z = 1, D_{calc} = 1.401 gcm^{-3}, F000 = 200, μ = 0.516 mm^{-1}, T = 100 K, θ_{max} = 52.746°, 14067 total reflections, 1497 with I_o>2σ(I_o), R_{int} = 0.0761, 1840 data, 110 parameters, 0 restraints, GooF = 1.048, R_1 = 0.0339 and wR_2 = 0.0848 [I_o>2σ(I_o)], R_1 = 0.0479 and wR_2 = 0.0954 (all reflections), 0.31<dΔρ<-0.35 eÅ^{-3}.

1.2.2 Complex H[24DiMePyNO]$_2$[Cl$_3$]·2[24DiMePyNO]

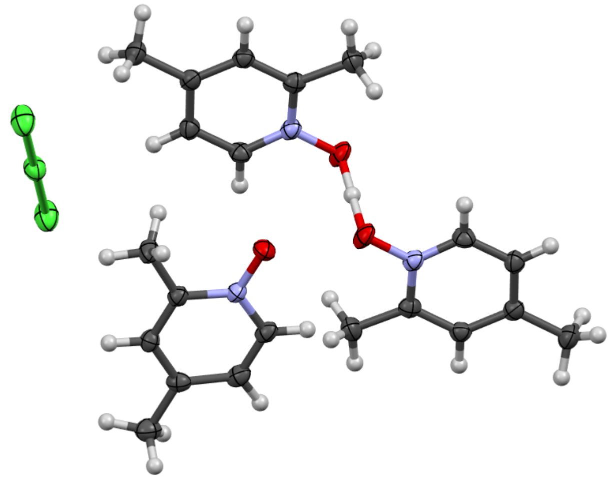

Figure S2. The X-ray crystal structure of **H[24DiMePyNO]$_2$[Cl$_3$]·2[24DiMePyNO]** with the thermal displacement parameter at 50% probability level. Colour Key: green = chlorine, blue = nitrogen, dark grey = carbon, white = hydrogen.

Crystal data for **H[24DiMePyNO]$_2$[Cl$_3$]·2[24DiMePyNO]**: CCDC-2383802. $C_{28}H_{37}Cl_3N_4O_4$, M = 599.96 g/mol, colorless plate, 0.261 × 0.093 × 0.053 mm^3, triclinic, space group $P\bar{1}$, a = 7.3618(17) Å, b = 8.291(2) Å, c = 13.265(3) Å, α = 85.903(9)°, β = 88.725(7)°, γ = 66.463(8)°, V = 740.4(3) Å^3, Z = 1, D_{calc} = 1.346 gcm^{-3}, F000 = 316, μ = 0.349 mm^{-1}, T = 100 K, θ_{max} = 52.914°, 3039 total reflections, 1972 with $I_o > 2\sigma(I_o)$, R_{int} = 0.1199, 3039 data, 183 parameters, 0 restraints, GooF = 1.049, R_1 = 0.0607 and wR_2 = 0.1420 [$I_o > 2\sigma(I_o)$], R_1 = 0.1003 and wR_2 = 0.1622 (all reflections), 0.38<dΔρ<-0.46 eÅ^{-3}, non-merohedric twin.

1.2.3. Complex H[2Me4OMePyNO]$_2$[Cl$_3$]

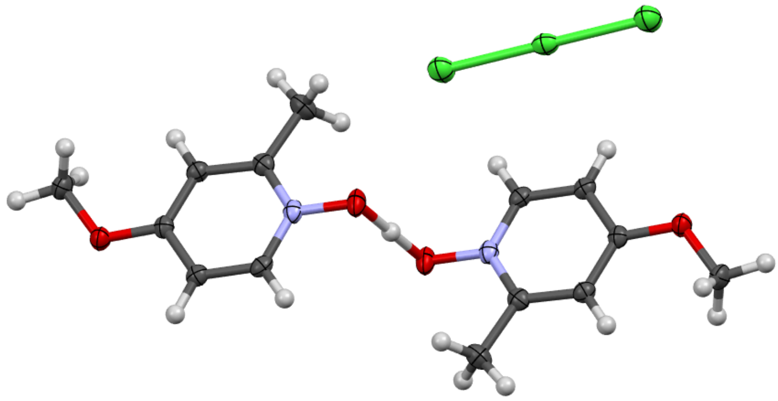

Figure S3. The X-ray crystal structure of **H[2Me4OMePyNO]$_2$[Cl$_3$]** with the thermal displacement parameter at 50% probability level. Colour Key: green = chlorine, blue = nitrogen, dark grey = carbon, white = hydrogen.

Crystal data for **H[2Me4OMePyNO]₂[Cl₃]**: CCDC-2383801. $C_{14}H_{19}Cl_3N_2O_4$, M = 385.66 g/mol, colorless block, 0.677 × 0.279 × 0.182 mm^3, monoclinic, space group *C2/c*, a = 11.8112(5) Å, b = 8.9258(3) Å, c = 17.6828(8) Å, α = 90°, β = 108.1780(10)°, γ = 90°, V = 1771.16(13) Å^3, Z = 4, D_{calc} = 1.446 gcm^{-3}, F000 = 800, μ = 0.537 mm^{-1}, T = 100 K, θ_{max} = 56.644 °, 2205 total reflections, 1955 with $I_o > 2\sigma(I_o)$, R_{int} = 0.0904, 2205 data, 109 parameters, 0 restraints, GooF = 1.119, R_1 = 0.0363 and wR_2 = 0.0909 [$I_o > 2\sigma(I_o)$], R_1 = 0.0414 and wR_2 = 0.0950 (all reflections), 0.60<dΔρ<-0.36 eÅ^{-3}, non-merohedric twin.

1.2.4. Complex H[2PhPyNO]₂[Cl₃]

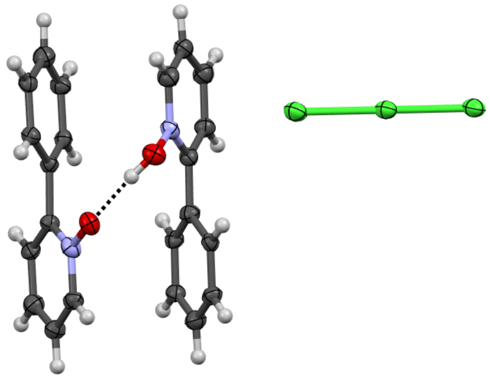

Figure S4. The X-ray crystal structure of **H[2PhPyNO][Cl₃]** with the thermal displacement parameter at 50% probability level. Colour Key: green = chlorine, blue = nitrogen, dark grey = carbon, white = hydrogen. The black broken line represents the hydrogen bond.

Crystal data for **H[2PhPyNO][Cl₃]**: CCDC-2383803. $C_{22}H_{19}Cl_3N_2O_2$, M = 449.74 g/mol, colorless needle, 0.387 × 0.045 × 0.025 mm^3, triclinic, space group $P\bar{1}$, a = 6.027(3) Å, b = 11.706(6) Å, c = 15.295(9) Å, α = 71.538(19)°, β = 81.375(19)°, γ = 89.527(14)°, V = 1011.1(10) Å^3, Z = 2, D_{calc} = 1.477 gcm^{-3}, F000 = 464.0, μ = 0.475 mm^{-1}, T = 100 K, θ_{max} = 50.786°, 3636 total reflections, 1278 with $I_o > 2\sigma(I_o)$, R_{int} = 0.2284, 3636 data, 264 parameters, 156 restraints, GooF = 1.086 , R_1 = 0.1321 and wR_2 = 0.2983 [$I_o > 2\sigma(I_o)$], R_1 = 0.3614 and wR_2 = 0.4380 (all reflections), 0.76<dΔρ<-0.79 eÅ^{-3}, non-merohedric twin.

1.2.5 Complex [PyNOH][Cl]

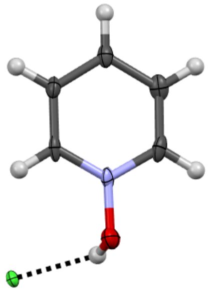

Figure S5. The X-ray crystal structure of **[PyNOH][Cl]** with the thermal displacement parameter at 50% probability level. Color Key: green = chlorine, blue = nitrogen, dark grey = carbon, white = hydrogen. Minor disordered fractions of chloride anions are omitted for viewing clarity. The black broken line represents the hydrogen bond.

Crystal data for **[PyNOH][Cl]**: CCDC-2383800. C_5H_6ClNO, M = 131.56 g/mol, colorless block, 0.128 × 0.12 × 0.08 mm^3, orthorhombic, space group $P2_12_12_1$, a = 7.2301(13) Å, b = 7.416(2) Å, c = 11.002(3)

Å, α = 90°, β = 90°, γ = 90°, V = 589.9(2) $Å^3$, Z = 4, D_{calc} = 1.481 gcm^{-3}, F000 = 272.0, μ = 0.537 mm^{-1}, T = 100 K, $θ_{max}$ = 50.652°, 674 total reflections, 621 with $I_o > 2σ(I_o)$, R_{int} = 0.1953, 674 data, 81 parameters, 18 restraints, GooF = 1.095, R_1 = 0.0931 and wR_2 = 0.2235 [$I_o > 2σ(I_o)$], R_1 = 0.1019 and wR_2 = 0.2327 (all reflections), 1.14<dΔρ<-1.03 $eÅ^{-3}$, non-merohedric twin.

1.2.6. Complex [26DiMePyNOH][Cl]

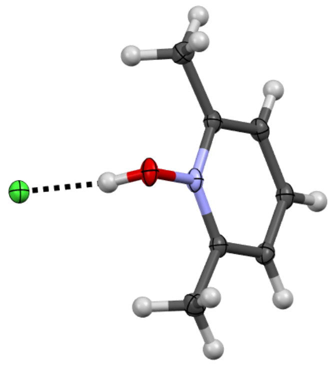

Figure S6. The X-ray crystal structure of **[26DiMePyNOH][Cl]** with the thermal displacement parameter at 50% probability level. Color Key: green = chlorine, blue = nitrogen, dark grey = carbon, white = hydrogen. The black broken line represents the hydrogen bond.

Crystal data for **[26DiMePyNOH][Cl]**: CCDC-2383798. $C_7H_{10}ClNO$, M = 159.61g/mol, colorless plate, 0.472 × 0.142 × 0.074 mm^3, monoclinic, space group $P2_1/m$, a = 6.2444(3) Å, b = 9.2125(4) Å, c = 6.7656(3) Å, α = 90°, β = 94.855(2)°, γ = 90°, V = 387.81(3) $Å^3$, Z = 2, D_{calc} = 1.367 gcm^{-3}, F000 = 168, μ = 0.421 mm^{-1}, T = 100 K, $θ_{max}$ = 52.704°, 8522 total reflections, 769 with $I_o > 2σ(I_o)$, R_{int} = 0.0434, 843 data, 54 parameters, 0 restraints, GooF = 1.116, R_1 = 0.0253 and wR_2 = 0.0621 [$I_o > 2σ(I_o)$], R_1 = 0.0312 and wR_2 = 0.0682 (all reflections), 0.25<dΔρ<-0.30 $eÅ^{-3}$.

1.2.7. Complex [2Me4OMePyNOH][Cl]

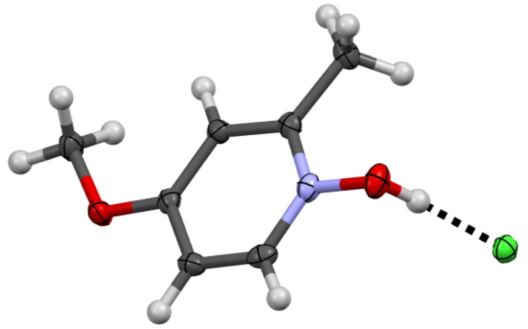

Figure S7. The X-ray crystal structure of **[2Me4OMePyNOH][Cl]** with the thermal displacement parameter at 50% probability level. Color Key: green = chlorine, blue = nitrogen, dark grey = carbon, white = hydrogen. The black broken line represents the hydrogen bond.

Crystal data for **[2Me4OMePyNOH][Cl]**: CCDC-2383806. $C_7H_{10}ClNO_2$, M = 175.61 g/mol, colorless block, 0.554 × 0.297 × 0.192 mm^3, triclinic, space group $P\bar{1}$, a = 7.7469(4) Å, b = 9.1814(5) Å, c = 13.0548(7) Å, α = 82.272(2)°, β = 80.701(2)°, γ = 66.100(2)°, V = 835.32(8) $Å^3$, Z = 4, D_{calc} = 1.396 gcm^{-3}, F000 = 368, μ = 0.407 mm^{-1}, T = 100 K, $θ_{max}$ = 52.744°, 34530 total reflections, 2926 with

$I_o > 2\sigma(I_o)$, R_{int} = 0.0507, 3416 data, 205 parameters, 0 restraints, GooF = 1.046, R_1 = 0.0269 and wR_2 = 0.0610 [$I_o > 2\sigma(I_o)$], R_1 = 0.0359 and wR_2 = 0.0665 (all reflections), 0.26<dΔρ<-0.27 eÅ^{-3}.

1.2.8. Complex [4tBuPyNOH][Cl]

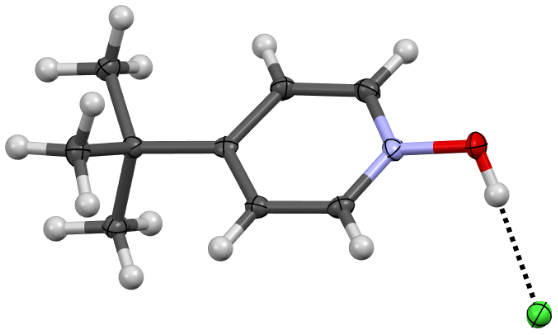

Figure S8. The X-ray crystal structure of **[4tBuPyNOH][Cl]** with the thermal displacement parameter at 50% probability level. Color Key: green = chlorine, blue = nitrogen, dark grey = carbon, white = hydrogen. The black broken line represents the hydrogen bond.

Crystal data for **[4tBuPyNOH][Cl]**: CCDC-2383805. $C_9H_{14}ClNO$, M = 187.66 g/mol, colorless block, 0.543 × 0.267 × 0.253 mm^3, orthorhombic, space group $P2_12_12_1$, a = 8.8661(5) Å, b = 10.3264(4) Å, c = 10.6277(6) Å, α = 90°, β = 90°, γ = 90°, V = 973.02(9) Å^3, Z = 4, D_{calc} = 1.281 gcm^{-3}, F000 = 400, μ = 0.346 mm^{-1}, T = 100 K, θ_{max} = 50.666°, 12603 total reflections, 1740 with $I_o > 2\sigma(I_o)$, R_{int} = 0.0328, 1776 data, 113 parameters, 0 restraints, GooF = 1.088, R_1 = 0.0204 and wR_2 = 0.0520 [$I_o > 2\sigma(I_o)$], R_1 = 0.0213 and wR_2 = 0.0528 (all reflections), 0.21<dΔρ<-0.16 eÅ^{-3}.

1.3. Halogen-bonded complexes

1.3.1. Complex Cl_2-PyNO

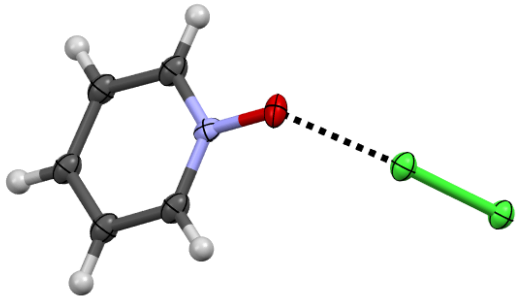

Figure S9. The X-ray crystal structure of **Cl_2-PyNO** with the thermal displacement parameter at 50% probability level. Color Key: green = chlorine, blue = nitrogen, dark grey = carbon, white = hydrogen. The black broken line represents the halogen bond. Disordered parts were omitted for viewing clarity.

Crystal data for **Cl_2-PyNO**: CCDC-2383807. $C_5H_5Cl_2NO$, M = 166.00 g/mol, colorless block, 0.554 × 0.231 × 0.134 mm^3, monoclinic, space group $C2/c$, a = 13.8648(10) Å, b = 13.7600(8) Å, c = 16.1649(15) Å, α = 90°, β = 115.221(2)°, γ = 90°, V = 2789.9(4) Å^3, Z = 16, D_{calc} = 1.581 gcm^{-3}, F000 = 1344, μ = 0.842 mm^{-1}, T = 100 K, θ_{max} = 50.69°, 28774 total reflections, 2651 with $I_o > 2\sigma(I_o)$, R_{int} = 0.0777, 2555 data, 140 parameters, 35 restraints, GooF = 1.282, R_1 = 0.0757 and wR_2 = 0.2459 [$I_o > 2\sigma(I_o)$], R_2 = 0.0874 and wR_2 = 0.2591 (all reflections), 0.69<dΔρ<-0.80 eÅ^{-3}.

1.3.2. Complex Cl_2-26DiMePyNO

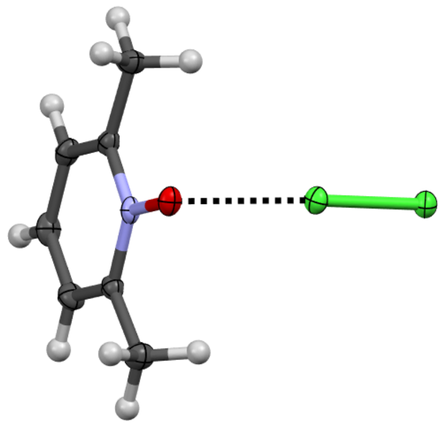

Figure S10. The X-ray crystal structure of **Cl_2-26DiMePyNO** with the thermal displacement parameter at 50% probability level. Color Key: green = chlorine, blue = nitrogen, dark grey = carbon, white = hydrogen. The black broken line represents the halogen bond.

Crystal data for **Cl_2-26DiMePyNO**: CCDC-2383799. $C_7H_9Cl_2NO$, M = 194.05 g/mol, colorless plate, 0.378 × 0.102 × 0.074 mm^3, monoclinic, space group $P2_1/m$, a = 6.9789(7) Å, b = 7.1228(6) Å, c = 8.6438(9) Å, α = 90°, β = 92.072(4)°, γ = 90°, V = 429.40(7) Å^3, Z = 2, D_{calc} = 1.501 gcm^{-3}, F000 = 200, μ = 0.696 mm^{-1}, T = 100 K, θ_{max} = 52.732°, 7419 total reflections, 795 with $I_o>2\sigma(I_o)$, R_{int} = 0.0431, 950 data, 64 parameters, 0 restraints, GooF = 1.261, R_1 = 0.0373 and wR_2 = 0.0756 [$I_o>2\sigma(I_o)$], R_1 = 0.0512 and wR_2 = 0.0887 (all reflections), 0.55<dΔρ<-0.48 eÅ^{-3}.

2. DFT studies

Table S1: Comparison of Cl_2, I_2 XB interaction energies, ΔE_{int} (kJ mol^{-1}) and HB interaction energies.

Complex	ΔE_{int}	XB/HB distance
PyNO···Cl_2	–29	2.446
2,6-Me_2-PyNO···Cl_2	–36	2.410
dioxane···Cl_2	–20	2.575
PyNO···I_2	–47	2.576
2,6-Me_2-PyNO···I_2	–55	2.555
PyNO···HCl	–59	1.493
2,6-Me_2-PyNOH···HCl	–71	1.426

Table S2: DFT computed ^{15}N shielding constants and chemical shifts.

	XB donor		XB acceptor	
	**Isotropic Shielding (ppm)**	**Chemical shift (ppm)**	**Isotropic Shielding (ppm)**	**Chemical shift (ppm)**
**NIS**	133	–283		
**PyNO**			–60	–90
**26DiMePyNO**			–55	–95
**Cl_2-PyNO**			–44	–106
**Cl_2-26DiMePyNO**			–39	–111
**I_2-PyNO**			–39	–111
**I_2-26DiMePyNO**			–34	–116
**NIS-PyNO**	97	-247	–40	–110
**NIS-26DiMePyNO**	90	-240	–34	–116

Optimized structures.xyz
This file cannot be rendered in this PDF. Please download the source file.
